# Point-of-care lactate in pediatric sepsis: a single-center observational study

**DOI:** 10.3389/fped.2026.1897796

**Published:** 2026-09-25

**Authors:** Abdulaziz A. Ajeebi, Nesrin A. Alharthy, Abdullatif G. Almalki, Mohammed K. Almutairi, Omar E. Tarabzoni, Hanan A. Alsuhibany, Razan M. Aldakheel, Hamad M. Alghanim, Tahir K. Hameed

**Affiliations:** 1 Pediatrics Emergency Department, King Abdullah Specialized Children’s Hospital, King Abdulaziz Medical City, Ministry of National Guard Health Affairs, Riyadh, Saudi Arabia; 2 Emergency Medical Services Department, College of Applied Medical Sciences, King Saud bin Abdulaziz University for Health Sciences, Riyadh, Saudi Arabia; 3 King Abdullah International Medical Research Center, Riyadh, Saudi Arabia; 4 Department of Pediatrics, King Abdullah Specialized Children’s Hospital, King Abdulaziz Medical City, Ministry of National Guard Health Affairs, Riyadh, Saudi Arabia; 5 College of Medicine, King Saud bin Abdulaziz University for Health Sciences, Riyadh, Saudi Arabia

**Keywords:** intensive care unit, lactic acid, pediatrics, point-of-care systems, sepsis

## Abstract

**Introduction:**

Lactate is a key biomarker in the assessment of sepsis, reflecting tissue hypoperfusion and correlating with disease severity and outcomes. However, reliance on standard laboratory testing may delay clinical decision-making in time-sensitive situations. Point-of-care (POC) lactate testing provides rapid bedside assessment, potentially facilitating earlier risk stratification and intervention. This study aimed to evaluate the predictive value of POC lactate for pediatric intensive care unit (PICU) admission and mortality in pediatric patients with sepsis.

**Methods:**

This was a retrospective observational study of children aged 3 months to 14 years presenting to the pediatric emergency department in a tertiary care children's hospital with a diagnosis of sepsis. Data were extracted from electronic medical records.

**Results:**

A total of 149 pediatric patients with sepsis were included, of whom 90 underwent POC lactate testing. Elevated POC lactate levels were observed in 38.9% of tested patients. Elevated POC lactate was significantly associated with PICU admission, as well as the need for mechanical ventilation and inotropic support. Receiver operating characteristic (ROC) analysis demonstrated modest but statistically significant predictive performance for PICU admission (AUC = 0.641), whereas no predictive value was observed for mortality (AUC = 0.544).

**Conclusions:**

Point-of-care lactate may serve as a practical supplementary biomarker that can facilitate early risk stratification by identifying children with sepsis at increased risk of PICU admission and requiring advanced interventions. Nevertheless, its modest discriminatory performance necessitates interpretation in conjunction with clinical assessment and additional biomarkers rather than as an independent predictor of sepsis severity.

## Introduction

Sepsis is one of the leading causes of mortality in children worldwide (1). Traditionally, it has been defined as a life-threatening condition secondary to an inflammatory or infectious process with organ dysfunction (2–4). The International Pediatric Sepsis Consensus Conference (IPSCC) in 2005 defined sepsis as a systemic inflammatory response syndrome (SIRS) in the presence of infection (5). In 2024, new criteria for pediatric sepsis were published. These criteria, known as the Phoenix Sepsis Score, consist of a 4-organ system model including criteria for cardio-vascular, respiratory, neurological, and coagulation dysfunction (1).

Although the mortality rate has been improving over recent decades, sepsis remains responsible for approximately 10%–20% of all deaths in the pediatric age group (6–8). Studies have shown that only minor improvements in sepsis outcomes have been observed, despite dramatic advances in medicine over the past decades (7, 9). Globally, sepsis is responsible for almost 25% of the mortality rate among patients admitted to the Pediatric Intensive Care Unit (PICU) (6). According to Gill et al., the United States loses almost 5,000 children annually secondary to sepsis (10). Locally, a recent study published at our quaternary care children's hospital found that amongst 189 PICU admissions due to sepsis, the overall mortality rate was 16.9% (11).

In addition to clinical assessment, many investigations are performed upon arrival in the Pediatric Emergency Department (ED) to evaluate the severity of sepsis. Lactate is one of the most essential and useful sepsis biomarkers that can be employed in the ED (12–14). Elevated serum lactate indicates tissue hypoperfusion secondary to sepsis. Hypoxia causes intracellular disturbance through the anaerobic pathway, and decreased lactate excretion in sepsis further leads to accumulation of lactate in the serum (15, 16).

Because of the time required to obtain central laboratory serum lactate results, Point-of-Care (POC) lactate testing represents a promising technique for clinicians to rapidly assess the severity of sepsis (17, 18). Early recognition of such critical presentations is crucial to prevent poor outcomes and mortality (19). Although POC lactate testing may guide clinicians toward appropriate management pathways, many physicians remain uncertain about its accuracy compared to central laboratory serum results. A systematic review published in 2013 regarding clinicians' attitudes toward POC blood testing identified inaccuracy as the most common barrier to adoption (20). Given the limited research on POC lactate in pediatric sepsis, we conducted this study to evaluate the accuracy of POC lactate in predicting sepsis severity and outcomes in children at a large quaternary care children's hospital in Riyadh, Saudi Arabia.

## Methods

### Study design

This study was a retrospective cohort study conducted at King Abdullah Specialized Children's Hospital (KASCH), a tertiary care pediatric center under the Ministry of National Guard Health Affairs in Riyadh, Saudi Arabia. The hospital comprises approximately 250 pediatric inpatient beds, and its pediatric emergency ED manages around 100,000 acute visits annually. Patients triaged as Canadian Triage and Acuity Scale (CTAS) levels 1 and 2 account for more than 15% of these ED visits. POC lactate testing is available at the bedside in the ED, performed by trained nursing staff and maintained by the institutional point-of-care center. The study evaluated patients who presented to the ED over a three-year period, from January 1, 2021 to December 31, 2023.

### Study population

The study population comprised pediatric patients aged 3 months to 14 years who presented to the emergency department (ED) and were clinically diagnosed and managed as having sepsis. Patient eligibility was determined based on the treating physician's documented clinical diagnosis of sepsis. Cases were identified through the electronic medical record using documented clinical diagnoses, with ICD-10 sepsis-related codes used to facilitate case retrieval. A manual chart review was subsequently performed to verify that all eligible patients met the predefined inclusion criteria and that the documented clinical presentation was consistent with the 2005 International Pediatric Sepsis Consensus Conference (IPSCC) definition of pediatric sepsis: systemic inflammatory response syndrome (SIRS) in the presence of suspected or confirmed infection (5). SIRS was defined by the presence of at least two of the following age-adjusted criteria, one of which was required to be either abnormal temperature or abnormal leukocyte count: (1) core temperature >38.5 °C or <36.0 °C; (2) tachycardia for age, or persistent unexplained bradycardia in infants younger than 1 year; (3) tachypnea for age or the need for mechanical ventilation due to an acute disease process; and (4) an abnormal leukocyte count for age or >10% immature neutrophils (band forms). The diagnosis of sepsis was established by the treating physician based on the overall clinical assessment, including patient history, physical examination, vital signs, and available laboratory findings, in accordance with routine emergency department practice. The 2005 IPSCC criteria were selected because they represented the accepted pediatric sepsis definition during the study period and rely on routinely available clinical and laboratory parameters used in the emergency department. Although the recently proposed Phoenix Sepsis Criteria (1) identify children with life-threatening organ dysfunction, their application requires additional laboratory investigations, including coagulation parameters such as D-dimer and fibrinogen, which were not routinely obtained for all patients during the initial ED evaluation. Following confirmation of eligibility, patients were classified into one of three groups according to the lactate testing strategy employed during their ED evaluation: (1) POC) lactate testing, (2) conventional laboratory serum lactate testing, or (3) no lactate testing. Group allocation reflected routine clinical practice and served as the basis for comparisons of patient characteristics, clinical management, and outcomes (Supplementary figure: patient flow chart).

Patients were excluded if they met any of the following criteria: age under 3 months, presence of cyanotic heart disease, active oncological conditions, known metabolic or genetic diseases, history of multiple PICU admissions, elective hospital admission, or postoperative status. Patients younger than 3 months of age were excluded from the study because they are managed using a different clinical protocol, particularly in cases of fever or clinically suspected sepsis. POC lactate concentrations were measured in the emergency department using the Nova StatStrip® Lactate Hospital Meter (Nova Biomedical, Waltham, MA, USA), a handheld *in vitro* diagnostic device designed for quantitative lactate determination in fresh venous or arterial whole blood. This device provides plasma-equivalent results comparable to laboratory methods. Blood samples were collected as part of routine clinical care and analyzed immediately at the bedside by trained emergency department healthcare professionals following the manufacturer's instructions. The device employs an electrochemical biosensor with lactate oxidase technology to generate quantitative lactate measurements from a small whole-blood sample. Routine quality control was conducted using manufacturer-provided control solutions in accordance with hospital policy, and patient testing was performed only after quality control results met the acceptable range. POC lactate results were automatically recorded in the electronic medical record and made available to the treating physician during the initial clinical assessment.

Central laboratory lactate measurements were conducted as part of routine patient care using the hospital's standard laboratory analyzer and accredited operating procedures. The choice between POC lactate testing, laboratory lactate testing, or both was determined by the treating physician, reflecting standard emergency department practice rather than a predefined study protocol. As a result, POC and laboratory lactate measurements were not consistently obtained as paired samples or at standardized time points. This study was therefore designed to evaluate the association of each testing strategy with clinical outcomes in routine practice, rather than to assess analytical agreement or equivalence between POC and laboratory lactate methods.

POC lactate values were analyzed as continuous variables (mmol/L) and as categorical variables using a predefined threshold of greater than 2.0 mmol/L (21). The research team used a standardized data collection sheet to gather relevant information. The extracted variables included patient demographics (e.g., age, gender), clinical presentation (e.g., fever, hypoactivity, difficulty breathing), hospital-related variables (e.g., admission status, PICU transfer) and bedside POC lactate levels.

### Statistical analysis

Statistical analyses were performed using Microsoft Excel and IBM SPSS Statistics. The primary outcome was PICU admission. Secondary outcomes included endotracheal intubation, inotrope/vasopressor requirement, hospital admission, culture positivity, and 30-day mortality, with mortality analyses considered exploratory because of the low event rate. Categorical variables were summarized as frequencies and percentages and compared using the Pearson chi-square test or Fisher's exact test when expected cell counts were small. Continuous variables were summarized as means ± standard deviations or medians with interquartile ranges (IQRs), as appropriate, and compared using the Mann–Whitney U test for non-normally distributed variables. Effect estimates with 95% confidence intervals were reported where appropriate. Receiver operating characteristic (ROC) curve analysis was performed to assess the ability of lactate measurements to discriminate between patients with and without the study outcomes. The area under the curve (AUC) and corresponding 95% confidence intervals were calculated. The optimal lactate cutoff for each outcome was determined using the Youden Index, which identifies the point on the ROC curve that provides the best balance between sensitivity and specificity. Sensitivity, specificity, positive predictive value, and negative predictive value corresponding to the optimal cutoff were calculated where applicable. Given the retrospective exploratory nature of the study, these thresholds were considered hypothesis-generating. No formal adjustment for multiple comparisons was performed, and the results should be interpreted accordingly. A two-sided *p*-value <0.05 was considered statistically significant. Table 1 shows the lactate analysis strategy.

**Table 1:** Statistical methods used to evaluate the association between lactate measurements and clinical outcomes.

Statistical Method	Purpose	Interpretation	Clinical Application
Pearson Chi-square Test/Fisher's Exact Test	Evaluates the association between elevated lactate (>2.0 mmol/L) and categorical clinical outcomes (e.g., PICU admission, intubation, vasopressor use, mortality).	A statistically significant result indicates that patients with lactate levels >2.0 mmol/L have a different frequency of the outcome compared with those with lactate ≤2.0 mmol/L.	Reflects the performance of a predefined clinically relevant lactate threshold used in routine practice.
Mann–Whitney U Test	Compares continuous lactate concentrations between patients with and without each clinical outcome.	A statistically significant result indicates that lactate concentrations differ between outcome groups regardless of any predefined cutoff.	Assesses whether absolute lactate concentration is associated with disease severity and clinical outcomes.
Receiver Operating Characteristic (ROC) Curve Analysis	Assesses the ability of lactate measurements to discriminate between patients with and without the outcome across all possible lactate thresholds.	The area under the ROC curve (AUC) quantifies overall discriminative performance, where an AUC of 0.5 indicates no discrimination and an AUC of 1.0 indicates perfect discrimination.	Evaluates the potential utility of lactate as a prognostic marker for clinical risk stratification.
Youden Index	Identifies the lactate cutoff that maximizes the combined sensitivity and specificity (Sensitivity + Specificity−1).	Provides the optimal statistical threshold for outcome prediction based on the study data.	The identified cutoff is exploratory and hypothesis-generating and should be externally validated before clinical implementation.

Fisher's exact test was used instead of the Pearson chi-square test when expected cell counts were fewer than five.

## Results

A total of 149 pediatric patients who presented to the ED for suspected sepsis were included in the study. The cohort was predominantly male (*n* = 110; 73.8%) and of Saudi nationality (*n* = 109; 73.2%). Forty-eight patients (32.2%) were 3–12 months of age. Co-morbidities were present in 59 (39.6%) of the patients. The most frequent clinical manifestations upon presentation were fever (68.2%), tachycardia (66.9%), and hypoactivity (45.9%). Regarding clinical outcomes, 113 patients (75.8%) required hospital admission, and 46 (30.9%) were admitted to the pediatric intensive care unit. The 30-day mortality rate for the cohort was 2.7%. POC lactate measurements were available for 90 patients (60.4%), while central serum lactate was measured in 49 patients (32.9%), and 10 patients (6.7%) were diagnosed with sepsis but had no lactate measured. Demographic and clinical characteristics are summarized in Table 2 and Figure 1.

**Table 2:** Demographic and clinical characteristics (*N* = 149).

Variable	N (%)	Variable	N (%)
Gender		Nationality	
Male	110 (73.8%)	Saudi	109 (73.2%)
Female	39 (26.2%)	Non-Saudi	40 (27.0%)
Age Group		Co-morbidities	
3 months – 12 months	48 (32.2%)	Yes	59 (39.6%)
13–36 months	43 (28.8%)	No	90 (60.4%)
4–5 years	16 (10.7%)	Hospital Admission	
6–12 years	36 (24.1%)	Yes	113 (75.8%)
13–14 years	6 (4%)	No	36 (24.2%)
PICU Admission		30-Day Mortality	
Yes	46 (30.9%)	Yes	4 (2.7%)
No	103 (69.1%)	No	145 (97.3%)
POC Lactate Available	90 (60.4%)	Serum Lactate Available	49 (32.9%)
Normal (≤2 mmol/L)	55 (61.1%)	Normal (≤2 mmol/L)	22 (44.9%)
Abnormal (>2 mmol/L)	35 (38.9%)	Abnormal (>2 mmol/L)	27 (55.1%)
Clinical Manifestations			
Fever	101 (68.2%)	Tachycardia	99 (66.9%)
Hypoactivity	68 (45.9%)	Difficulty Breathing	49 (33.1%)
Vomiting	47 (31.8%)	Lethargic	41 (27.7%)
Convulsion	35 (23.6%)	Diarrhea	27 (18.2%)
White blood cell count			
Mean	9.9	25 Percentiles	6.125
Median	7.7	50 Percentiles	7.4
Mode	6.4	75 Percentiles	11.9

POC, point-of-care; PICU, pediatric intensive care unit; Values expressed as *N* (%).

**Figure 1:**
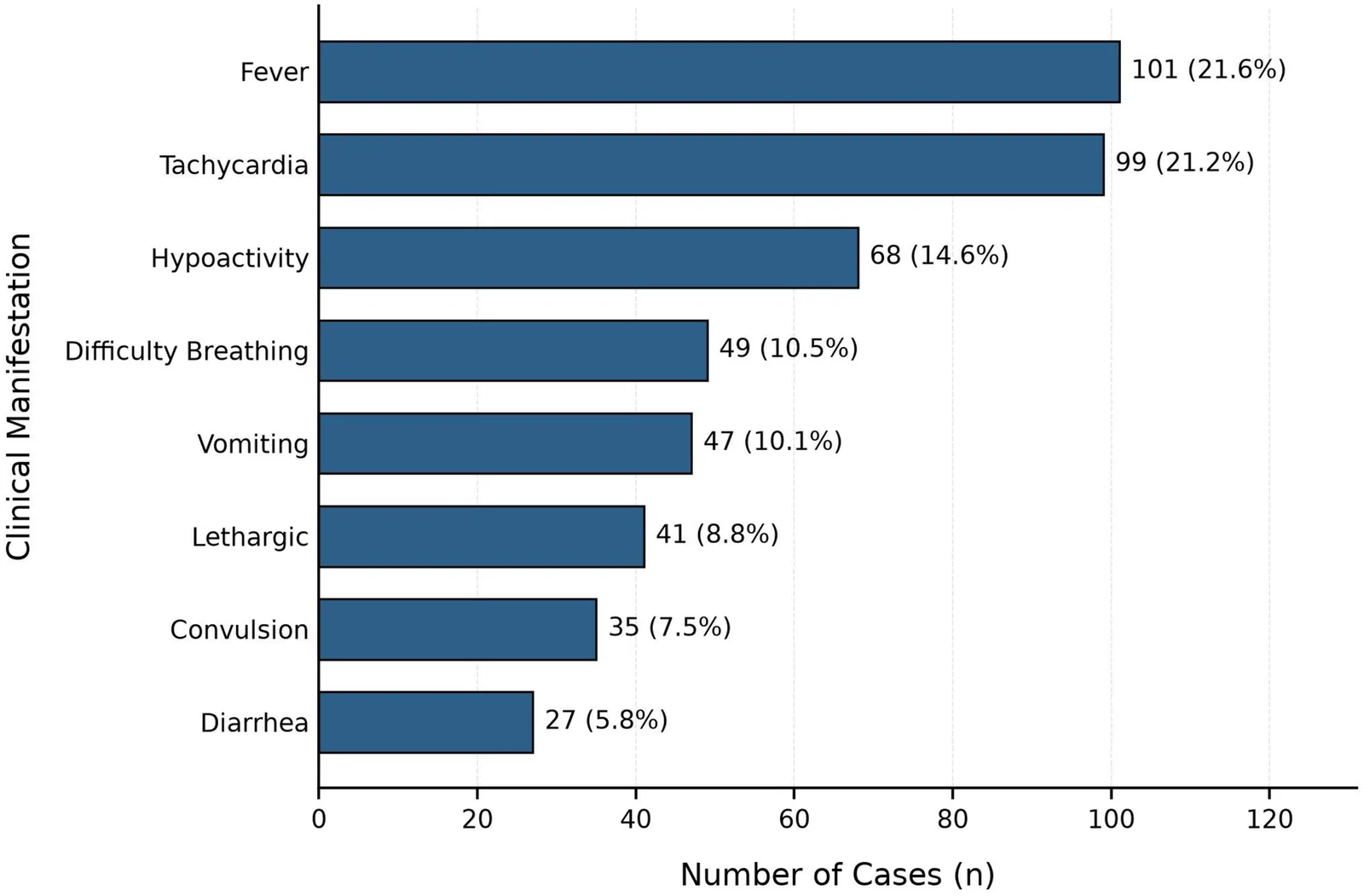
Clinical manifestations at presentation of pediatric patients diagnosed with sepsis.

Among the 90 patients with POC lactate measurements, the median lactate level was 1.80 mmol/L (IQR 1.24–2.70). Elevated POC lactate (>2 mmol/L) was observed in 35 patients (38.9%). There was a statistically significant association between elevated POC lactate and the requirement for PICU admission (*χ*^2^ = 7.42, *p* = 0.00645). When evaluated as a continuous variable, median POC lactate was significantly higher in PICU-admitted patients than in non-PICU patients (Mann–Whitney U test, *p* = 0.024). Furthermore, POC lactate as a continuous variable was significantly associated with the need for endotracheal intubation (Mann–Whitney *p* = 0.018) and the requirement for inotrope or vasopressor support (Mann–Whitney *p* = 0.012). POC lactate did not show a significant association with 30-day mortality, hospital admission, fluid resuscitation, or culture positivity (all *p* > 0.05).

Among the 49 patients with central serum lactate measurements, the median level was 2.26 mmol/L (IQR 1.25–4.00), with 55.1% (*n* = 27) exhibiting levels >2 mmol/L. Serum lactate did not demonstrate a statistically significant association with PICU admission as a binary variable (*χ*^2^ = 0.93, *p* = 0.336) or as a continuous variable (Mann–Whitney *p* = 0.773). Similarly, serum lactate was not significantly associated with intubation, inotrope requirement, or mortality. The associations between both lactate measures and all clinical outcomes are detailed in Tables 3, 4.

**Table 3:** Diagnostic performance of point-of-care versus serum lactate for predicting PICU admission.

	Value (%)	95% Confidence Interval
Point-of-care lactate (*n* = 90)		
Sensitivity (abnormal POC in PICU patients)	21/38 (55.3%)	39.7–69.9%
Specificity (normal POC in non-PICU patients)	38/52 (73.1%)	59.7–83.2%
Positive Predictive Value (PICU admission if POC abnormal)	21/35 (60%)	43.6–74.4%
Negative Predictive Value (non-PICU if POC normal)	38/55 (69.1%)	56.0–79.7%
Chi-Square Test	*χ*^2^ = 7.42 *p* = 0.00645*	
Serum lactate (*n* = 49)		
Sensitivity (abnormal serum lacate in PICU patients)	16/26 (61.5%)	42.5–77.6%
Specificity (normal serum lacate in non-PICU patients)	12/23 (52.2%)	33.0–70.8%
Positive Predictive Value (PICU admission if serum lactate abnormal)	16/27 (59.3%)	40.7–75.5%
Negative Predictive Value (non- PICU if serum lactate normal)	12/22 (54.5%)	34.7–73.1%
Chi-Square Test	*χ*2 = 0.93, *p* = 0.336	

* p *<* *0.05.*

**Table 4:** Association of POC and serum lactate with clinical outcomes.

Outcome	POC *χ*²	POC *p* (*χ*²)	POC *p* (MW)	POC AUC (95% CI)	POC ROC *p*	Serum χ²	Serum *p* (χ²)	Serum *p* (MW)	Serum AUC (95% CI)	Serum ROC *p*
PICU Admission	5.64	0.018*	0.024*	0.641 (0.523– 0.759)	0.020*	0.06	0.799	0.773	0.525 (0.360– 0.690)	0.764
Intubation	3.48	0.062	0.018*	0.655 (0.530– 0.779)	0.015*	1.39	0.239	0.419	0.428 (0.255– 0.601)	0.413
Inotropes	2.32	0.128	0.012*	0.734 (0.558– 0.911)	0.009*	0.00	1.000	0.533	0.419 (0.183– 0.654)	0.498
Cardiac Arrest	0.00	1.000	0.162	0.709 (0.417– 1.000)	0.161	0.05	0.822	0.881	0.470 (0.136– 0.805)	0.862
30-Day Mortality	0.00	0.976	0.774	0.544 (0.247– 0.841)	0.771	0.62	0.430	0.332	0.349 (0.092– 0.606)	0.251
Fluid Resuscitation	0.00	1.000	0.435	0.452 (0.331– 0.572)	0.431	2.38	0.123	0.037*	0.312 (0.160– 0.465)	0.016*

χ2: Pearson chi-square test (binary >2 mmol/L cutoff); MW: Mann–Whitney U test (continuous lactate); AUC: area under the ROC Curve (95% CI by Hanley-McNeil method); * *p* < 0.05 (statistically significant, shown in bold).

ROC curve analysis was performed to evaluate the discriminative ability of lactate levels. POC lactate demonstrated modest but statistically significant discrimination for PICU admission (AUC = 0.641, 95% CI 0.523–0.759, *p* = 0.020), intubation requirement (AUC = 0.655, 95% CI 0.530–0.779, *p* = 0.015), and acceptable discrimination for inotrope/vasopressor use (AUC = 0.734, 95% CI 0.558–0.911, *p* = 0.009). The optimal POC lactate cutoff for predicting PICU admission by the Youden Index was 2.30 mmol/L.

Conversely, serum lactate did not demonstrate significant discriminative ability for PICU admission (AUC = 0.525, 95% CI 0.360–0.690, *p* = 0.764) or any other major clinical outcome. Neither POC nor serum lactate showed discriminative ability for 30-day mortality. ROC analysis results are summarized in Table 5 and Figure 2.

**Table 5:** ROC curve analysis: discriminative ability of lactate for clinical outcomes.

Outcome	Method	AUC	95% CI	*p*-value	Optimal Cutoff	Sensitivity	Specificity	Interpretation
PICU Admission	POC Lactate	0.641	0.523– 0.759	0.020*	2.30 mmol/L	63.0%	65.0%	Modest
PICU Admission	Serum Lactate	0.525	0.360– 0.690	0.764	2.26 mmol/L	54.2%	52.0%	No discrimination
Intubation	POC Lactate	0.655	0.530– 0.779	0.015*	1.80 mmol/L	72.1%	57.0%	Modest
Inotropes	POC Lactate	0.734	0.558– 0.911	0.009*	2.70 mmol/L	72.7%	70.0%	Acceptable
30-Day Mortality	POC Lactate	0.544	0.247– 0.841	0.771	—	—	—	No discrimination

AUC, area under the curve; CI: confidence interval (Hanley-McNeil method); Optimal Cutoff: Youden Index; * *p* < 0.05 (statistically significant, shown in bold).; Interpretation: Excellent ≥0.80, Acceptable 0.70–0.79, Modest 0.60–0.69, No discrimination <0.60.

**Figure 2:**
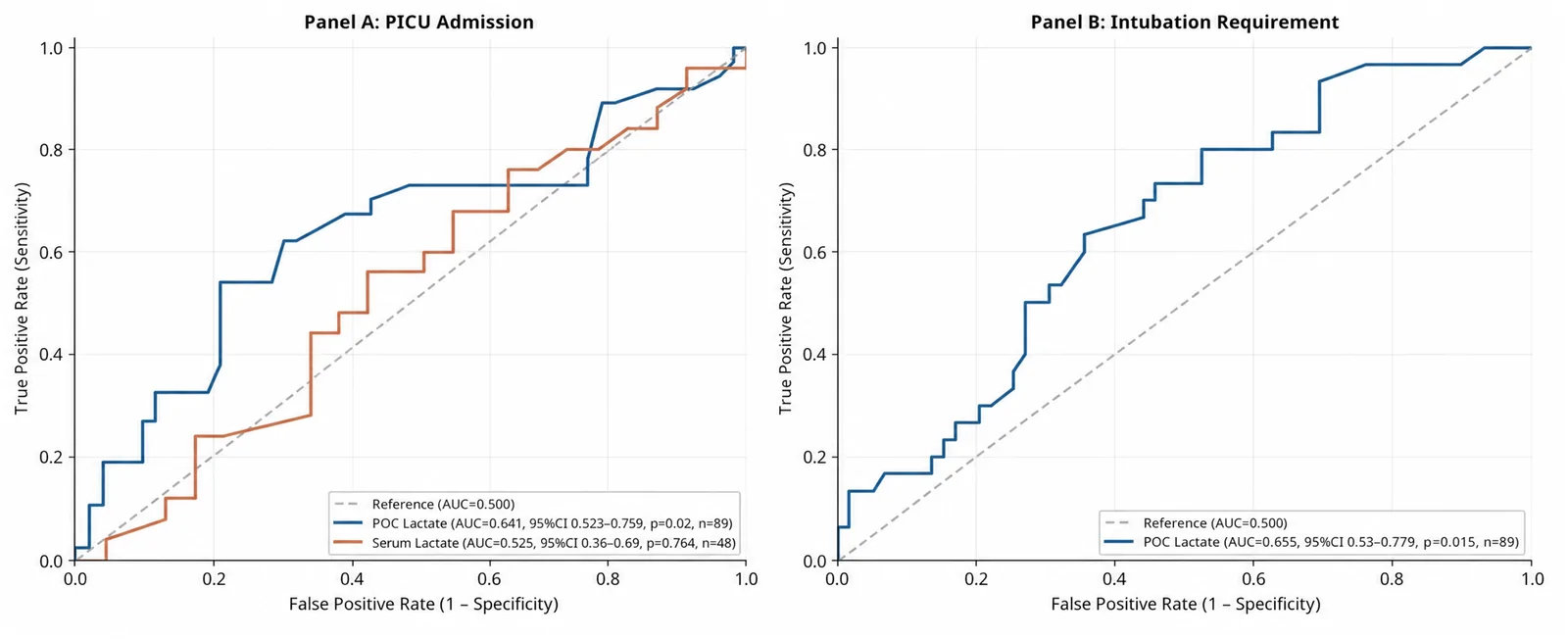
ROC curves for discriminative ability of lactate. Panel **(A)**: PICU admission; Panel **(B)**: intubation requirement.

## Discussion

The current study evaluated point-of-care (POC) lactate testing as a predictor of clinical outcomes including PICU admission, intubation, inotrope use, and 30-day mortality in pediatric sepsis patients presenting to the emergency department. Elevated POC lactate levels (>2 mmol/L) were significantly associated with an increased likelihood of PICU admission. However, due to the modest discriminatory performance (AUC = 0.641) and the retrospective study design, these results indicate an unadjusted association rather than independent predictive value. These results align with existing literature supporting POC lactate as a reliable biomarker for risk stratification in sepsis (17, 18).

The discriminative ability of POC lactate for PICU admission was modest (AUC = 0.641, 95% CI 0.523– 0.759, *p* = 0.020), and acceptable for inotrope/vasopressor requirement (AUC = 0.734, *p* = 0.009). These findings are consistent with published adult and pediatric studies. Shapiro et al. (22) demonstrated that bedside POC lactate testing could correlate with clinical outcomes in emergency department patients with suspected infection. Gicheru et al. (23) similarly reported that initial POC lactate is a useful tool in terms of prediction of outcomes of adult patients suspected with sepsis and septic shock, establishing a cutoff of ≥3.5 mmol/L.

The study findings indicate that point-of-care (POC) lactate testing may offer a rapid measurement to support early clinical assessment in the emergency department. However, due to the retrospective study design and the modest discriminatory performance observed, these results demonstrate an association rather than independent predictive utility. The clinical value of POC lactate as a component of early risk stratification should be confirmed in prospective studies before routine incorporation into clinical decision-making or sepsis management protocols is recommended.

Despite its utility in predicting PICU admission and inotropic support requirement, POC lactate in our study did not show significant discriminative ability for predicting 30-day mortality (AUC = 0.544, *p* = 0.771). This finding is likely attributable to the very low overall mortality rate in our cohort, which severely limits the statistical power to detect a significant association. This low mortality rate is consistent with recent epidemiological data from developed healthcare settings, where pediatric sepsis mortality often ranges between 2% and 5% (4, 7). In contrast, a large study from the United States (24) demonstrated that elevated early lactate levels (>4.0 mmol/L) are significantly associated with a threefold increase in 30-day mortality in children with clinically suspected sepsis. This discrepancy highlights the need for larger studies to capture sufficient adverse events for robust mortality prediction. Combined biomarker approaches, such as the lactate/albumin ratio, have been proposed to enhance prognostic performance beyond standalone lactate in pediatric infections (25). Similarly, the procalcitonin-to-albumin ratio has shown promise as a mortality biomarker in sepsis (26), further supporting the evolving role of combined approaches in sepsis prognosis.

A notable finding in our study was the absence of significant discriminative ability of central serum lactate across most clinical outcomes, including PICU admission, except for fluid resuscitation serum lactate showed a weak and paradoxical association with the secondary outcome of fluid resuscitation. This contrasts with existing literature supporting serum lactate as a predictor of illness severity (15, 16). The lack of significance was likely influenced by the smaller subset of patients who underwent central serum testing (*n* = 49) compared to POC testing (*n* = 90), limiting statistical power. Additionally, delays in obtaining central laboratory results may weaken the correlation between the measured value and the acute clinical decision to admit to the PICU. These findings suggest that in resource-limited or time-sensitive settings, POC lactate may offer a more practical and equally informative alternative to central serum lactate.

We also found that elevated POC lactate was significantly associated with an increased requirement for endotracheal intubation. This highlights the link between metabolic stress, systemic inflammation, and the need for advanced airway support. This is consistent with studies indicating that elevated lactate levels, reflecting tissue hypoxia and accelerated anaerobic glycolysis, often precede severe respiratory compromise and the necessity for mechanical ventilation in septic patients (27, 28). A consistent pattern emerged across multiple outcomes: categorical analysis using the predefined threshold of >2.0 mmol/L produced results that differed from those obtained through continuous analysis of lactate values. This discrepancy was most pronounced in the intubation outcome. Continuous POC lactate values differed significantly between intubated and non-intubated patients, whereas the categorical threshold did not significantly predict intubation status. A similar discordance was identified for fluid resuscitation, as serum lactate was significantly associated in the Mann–Whitney test but not in the chi-square analysis.

Several important limitations must be acknowledged when interpreting our findings. As a retrospective single-center cohort study, the analysis is inherently subject to selection bias and confounding. The lactate testing strategy (point-of-care, laboratory, or none) was determined by treating physicians during routine clinical practice rather than through a standardized protocol, potentially introducing systematic differences between patient groups that were not adjusted for in the analysis. Lactate measurements were not obtained as paired samples or at standardized time points, limiting comparability across groups and preventing assessment of temporal relationships with clinical deterioration. The absence of multivariable statistical adjustment for key confounders, such as baseline disease severity, shock indicators, and SIRS component values, may have permitted unmeasured differences between testing strategy groups to independently influence outcomes. No formal correction for multiple comparisons was applied despite the use of numerous statistical tests, increasing the risk of Type I error and the identification of spurious associations. Therefore, results should be interpreted as exploratory and hypothesis-generating rather than confirmatory. PICU admission criteria and inotrope or vasopressor use thresholds were not formally defined, reflecting institutional practice patterns that may vary across centers and limit generalizability. The study population was restricted to patients aged 3 months to 14 years with multiple exclusion criteria (cyanotic heart disease, active oncology, metabolic disorders, multiple prior PICU admissions), which may have selected for less severely ill sepsis patients and limits applicability to broader pediatric populations. Although the 2005 IPSCC sepsis definition was appropriate for the study period, verification relied on documented clinical diagnoses rather than independent researcher-applied criteria, introducing potential for diagnostic misclassification. The predefined lactate threshold of >2.0 mmol/L and optimal cutoffs derived from Youden Index optimization may be overfitted to this specific cohort and require external validation before clinical application. Finally, as the study was conducted at a single tertiary care center in Saudi Arabia, findings may not be generalizable to other healthcare systems, populations, or resource settings. Collectively, these limitations indicate that the findings should be considered exploratory and hypothesis-generating, with external validation required prior to implementation in clinical practice.

## Conclusion

POC lactate is a practical, readily accessible biomarker in the pediatric ED. It serves as a predictor for PICU admission, the need for advanced airway support, and inotrope requirement in children with sepsis. Our findings demonstrate that POC lactate provides limited, threshold-independent discriminatory value for multiple sepsis-related outcomes, while serum lactate shows minimal utility within this cohort. The discordance observed between categorical and continuous analyses, combined with fair-to-poor AUC values, indicates that lactate is not a reliable standalone predictor of outcome and is not adequately represented by the predefined threshold of greater than 2.0 mmol/L. These results align with recent literature, which underscores that lactate represents only one component of sepsis severity assessment and should be interpreted in conjunction with clinical judgment, hemodynamic parameters, organ function markers, and serial trends, rather than as an isolated measurement.

## Data Availability

The raw data supporting the conclusions of this article will be made available by the authors, without undue reservation.
